# Flexible Strain Sensors Based on Bionic Parallel Vein-like Structures for Human Motion Monitoring

**DOI:** 10.3390/s24020468

**Published:** 2024-01-12

**Authors:** Boshuo Yin, Furong Liu, Qingyuan Chen, Ming Liu, Feiying Wang

**Affiliations:** 1 Key Laboratory of Trans-Scale Laser Manufacturing (Beijing University of Technology), Ministry of Education, Beijing 100124, China; 2 Beijing Engineering Research Center of Laser Technology, Beijing University of Technology, Beijing 100124, China

**Keywords:** microstructure, flexible strain sensors, stretching, human motion, pressure-sensitive sensors

## Abstract

In recent years, strain sensors have penetrated various fields. The capability of sensors to convert physical signals into electrical signals is of great importance in healthcare. However, it is still challenging to obtain sensors with high sensitivity, large operating range and low cost. In this paper, a stretchable strain sensor made of a double-layer conductive network, including a biomimetic multilayer graphene-Ecoflex (MLG-Ecoflex) substrate and a multilayer graphene-carbon nanotube (MLG-CNT) composite up-layer was developed. The combined action of the two layers led to an excellent performance with an operating range of up to 580% as well as a high sensitivity (gauge factor (GF_max_) of 1517.94). In addition, a pressure sensor was further designed using the bionic vein-like structure with a multi-layer stacking of MLG-Ecoflex/MLG-CNT/MLG-Ecoflex to obtain a relatively high deformation along the direction of thickness. The device presented a high sensing performance (up to a sensitivity of 0.344 kPa^−1^) capable of monitoring small movements of the human body such as vocalizations and gestures. The good performance of the sensors together with a simple fabrication procedure (flip-molding) make it of potential use for some applications, for example human health monitoring and other areas of human interaction.

## 1. Introduction

With the growing focus on human health, it is increasingly vital to monitor movement, breathing and other physiological characteristics. By tracking and recording the movement information of human joints, it is possible to determine the amplitude and orientation of human movements, and, furthermore, to diagnose the health status of joints or muscle groups, which is of great significance in the medical field [1,2,3,4]. On the other hand, if various physiological signals such as micro-expressions, breathing or vocalizations can be accurately captured, in addition to effectively diagnosing humans’ physical condition, devices such as electronic prostheses can be prepared by combining them with robotics technology to bring some convenience to people with disabilities [5,6,7]. The stated requirements necessitate the utilization of sensors with fast response rates, high sensitivity and great flexibility to accurately detect instantaneous large deformations of the human body and capture subtle movements. However, the majority of conventional strain sensing elements employ semiconductors and metallic materials, which fail to adhere to the latest trend of strain sensors and the requirements of health detection devices for humans due to their poor biocompatibility, inflexibility, inconvenience and limited sensing range (commonly <5%) [8]. Therefore, researchers have used silicone-based elastomers (e.g., polydimethylsiloxane, Ecoflex) [9,10,11,12,13] and polymer elastomers (e.g., hydrogels and polyurethane (PU)) [14,15,16], one-dimensional nanomaterials such as metal nanowires [17,18] and carbon nanotubes [19,20] and flaky two-dimensional materials such as graphene nanosheets [21,22,23] and MXene [24,25] to constitute conductive films and to design and fabricate stretchable and highly sensitive flexible strain sensors. Lee et al. [18]. fabricated a crack sensor based on AgNW (Silver Nanowires)/PDMS by depositing AgNWs on PDMS with a maximum stretch of up to 100% and a sensitivity of 30. Wang et al. [23] reported a flexible sensor based on overlapping reduced graphene oxide flakes with a sensing range five times greater than that of a strain sensor based on a single graphene flake. In addition, the conductive film can be structurally designed to have a certain microstructure, and the deformation of the microstructure and the change of the contact area can be utilized for strain sensing. Huang et al. [26] covered carbon nanotubes on PDMS with pyramid microstructure and assembled a micro-pressure sensor with ultrahigh sensitivity and flexibility. Park et al. [27] designed and fabricated CNT-PDMS arrays with interlocking structures of CNT-PDMS, where, when two conductive interlocking microdome arrays were placed in a stack relative to each other, the contact area between the microdomes was drastically reduced during stretching, resulting in a significant increase in resistance, which, in turn, improved the sensitivity of this sensor.

However, the microstructured films mentioned above were fabricated by replicating the structure of patterned silicon templates produced by a photolithographic technique, which is costly and time-consuming. Instead, we found that a regular, parallel vein-like structure exists naturally in banana leaves, and we incorporated this microstructure into the sensor. A flexible substrate was prepared by flip-molding a naturally occurring plantain leaf, which formed the lower conductive structure. The substrate is capable of adapting to strain through stretching and recovery of its parallel vein-like microstructure [28]. An upper conductive structure layer was produced by multilayer graphene (MLG) and carbon nanotube (CNT) composites. Consequently, we fabricated a flexible strain sensor with a double-layer conductive network with a bionic parallel vein-like structure. The sensor has a wide sensing range of up to 580%, as well as ultra-high sensitivity and fast response characteristics. Furthermore, an MLG-Ecoflex/MLG-CNT/MLG-Ecoflex multilayer stacked pressure sensor was prepared using a bionic parallel vein-like structure. When subjected to pressure, the sensor increases the conductive channels due to the deformation of the microstructure, resulting in an increase in current change. The pressure sensors exhibit excellent linearity and a sensitivity of 0.344 kPa^−1^.

## 2. Experimental Section

**Fabrication of conductive films with bionic parallel vein-like structures.** First, the intact relatively flat banana leaf piece was chosen and cut into a 2 cm × 2 cm standard rectangular blade; the surface of the blade was rinsed with deionized water and, rinsed with ethanol and then blown dry with a hairdryer to obtain the template piece of banana leaf. The leaf blade was then pasted flat into the rectangular silicone mold using double-sided adhesive tape. Next, Ecoflex silica gel (Smooth On Ecoflex 00–30, Smooth-On, Inc., Macungie, PA, USA) was mixed in a 1:1 weight ratio of part A to part B. A measured amount of MLG (multi-layer graphene) was added to the gel mixture and thoroughly stirred. The mixture was then vacuum processed to remove any trapped air bubbles. It was poured into the rectangular silicone mold. To ensure the integrity of the microstructure replica, any air bubbles between the mold and the silica gel were removed in a vacuum drying oven. The MLG-Ecoflex layer was peeled off from the blade after curing at a constant temperature of 75 °C for 30 min. This resulted in the MLG-Ecoflex substrate layer with parallel-vein microstructures serving as the lower conductive layer. In the third step, CNTs were immersed in anhydrous ethanol and ultrasonicated for 1 h. MLG was then added to the solution containing CNTs and ultrasonicated for 2 h. The mass ratio of MLG to CNTs was chosen based on the desired standard, ranging from 1 to 9:1. After stirring for 30 min, the mixture was placed in a drying oven at 70 °C for 24 h, resulting in the MLG-CNT composite material. Finally, the substrate layer obtained in the second step was pre-stretched. The MLG-CNT composite material obtained in the third step was coated onto the pre-stretched substrate. This process yielded a conductive structure with a surface microstructure resembling parallel veins.

**Fabrication of strain sensors.** Firstly, after mixing part A and part B, Ecoflex was poured on a clean glass sheet. The spin coater was used to coat it uniformly, and it was cured at a constant temperature of 75 °C. The cured film was then cut into a size of 2 cm × 2 cm to obtain the upper encapsulation layer of the sensor. Secondly, the conductive silver glue was used to paste the wires on the left and right ends of the upper conductive layer, and it was dried at room temperature for 1 h. Finally, the upper encapsulation layer was overlapped with the conductive film layer to obtain a flexible stretching strain sensor. 

**Experimental characterization.** The surface micro-morphology of the upper and lower conductive layers as well as the surface morphology of the conductive layer under different degrees of strain were characterized by field emission electron scanning electron microscopy (SEM) (IT700, JEOL Ltd., Tokyo, Japan).

An electrochemical workstation (PMC CHS08A + WAVEDRIVE20, AMETEK, Berwyn, PA, USA) and a motorized stepper were used to build a sensor performance test platform to measure the I-t curves of the strain sensors in real time. The motorized displacement stage was connected to both ends of the samples, and uniformly increasing strains as well as stretch–release cycles were applied to the samples to test the strain sensing properties and capabilities of the samples.

## 3. Results and Discussion

### 3.1. Surface Microstructure Characterisation

**Figure 1:**
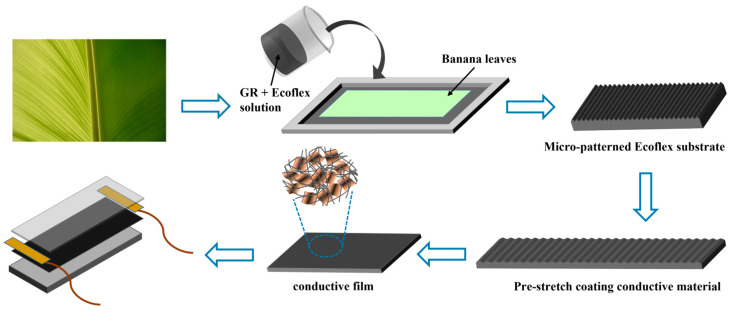
Schematic diagram of the fabrication process of flexible strain sensors.

**Figure 2:**
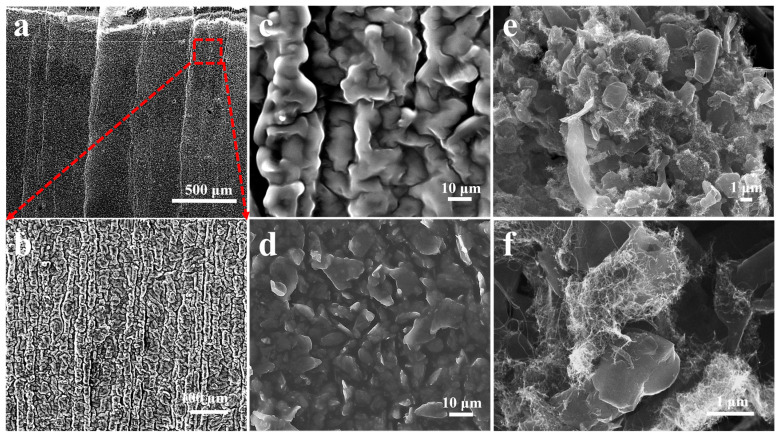
Microscopic morphology of the upper and lower conductive layers. (**a**,**b**) Low and high-magnification scanning electron microscopy (SEM). (**c**,**d**) Surfaces of the double-layer conductive structure before and after coating with conductive powder. (**e**,**f**) High magnification SEM of MLG-CNT.

The process of preparing this strain sensor is illustrated in Figure 1. The MLG-Ecoflex flexible substrate with bionic parallel veins was prepared as a template for the lower conductive layer using washed banana leaves, and its microscopic morphology is shown in Figure 2a,b. On the flexible substrate, the MLG-CNT composites were coated on the microstructures by the experimental means of pre-stretching, so that the MLG-CNT composites were uniformly coated on the surface of the substrate, which was prepared as a flexible conductive film. It exhibits the microstructure morphology of the surface prior and subsequent to coating, which clearly illustrate the coverage of the parallel vein-like microstructures by MLG-CNT composites (Figure 2c,d). In addition, the minute ridge-and-groove-like structure on the surface of the flexible substrate functions as a pinning effect on the upper conductive layer powder, improving the bonding between the two. The histomorphology of the upper conductive layer composite of MLG-CNT was analyzed under a high magnification microscope, as presented in Figure 2e,f. It can be inferred that the CNTs have adhered to the surface of the graphene sheet to wrap it.

### 3.2. Sensing Performance Analysis

**Figure 3:**
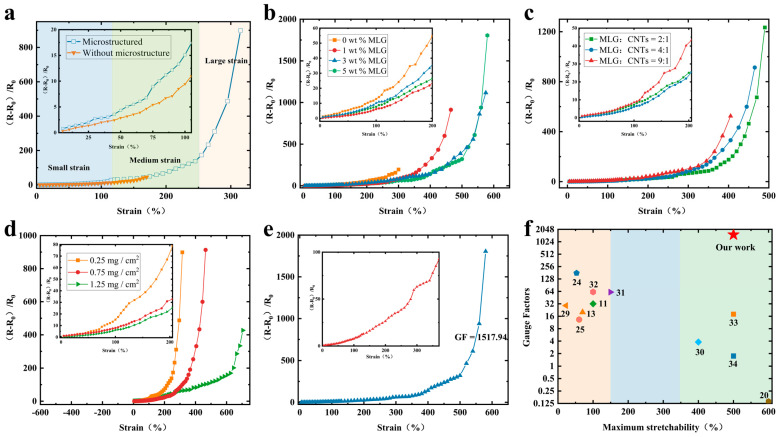
(**a**) Sensing characteristic curve of the stretch strain sensor with and without microstructure. Relative resistive-strain variation curves of strain sensors (**b**) with MLG concentrations of 0 wt%, 1 wt%, 3 wt%, and 5 wt% in the Ecoflex polymer (lower conductive layer), and (**c**) with 2:1, 4:1 and 9:1 mass ratios of MLG to CNTs in the upper conductive layer. (**d**) At different unit contents of the upper conductive layer composites under MLG to CNT mass ratio of 4:1. (**e**) Sensing characteristics curve of the best-performing stretch strain sensor. (**f**) Comparison with reported research works.

The study systematically investigated and analyzed the electromechanical properties of flexible strain sensors with bionic parallel vein-like structure based on a double-layer conductive network. The relative resistance of the sensor varies with the amount of stretch strain (Figure 3a–e), and the sensitivity (GF) of the sensor is determined by the slope of the curve of the relative resistance with the strain. Accordingly, $GF=\frac{R-R_{0}}{R_{0}\varepsilon }$, where $\frac{R-R_{0}}{R_{0}}$ represents the value of the relative resistance, *R*_0_ represents the initial resistance, *R* is the resistance under stretching and *ε* is the change of stretching. The strain–resistance curves of the flexible strain sensors are divided into three linear regions within the sensing range (Figure 3a), which are related to the changes in the microstructure of the surface of the double-layer conductive network during stretching as well as the underlying mechanisms. Additionally, a series of control experiments were conducted to test the sensing capabilities of both sensors with and without microstructures (see Figure 3a). It indicated that although the stretchability of the microstructure-free sensor itself was well above 170% strain, it was an ineffective operating range at stretch rates above 170%. The results showed that the introduction of microstructures widened the operating range of the sensor and improved its sensitivity. 

As shown in Figure 3b, the effect of different MLG concentrations (conductive polymers with 0 wt%, 1 wt%, 3 wt%, 5 wt% MLG concentration) in the substrate layer (MLG-Ecoflex) on the relative resistive-strain variation curves of the sensors was investigated. The sensing curves exhibit noticeable distinctions across different MLG concentrations in the substrate layer. As the MLG concentration increases, the strain range of the sensor widens. However, when the concentration surpasses an optimum level, the sensor’s sensitivity decreases at the same strain due to the excessive number of parallel conductive channels that hinder conductivity change. The sensor having a substrate MLG concentration of 1 wt% exhibits the least relative resistance alteration within the range of 250% sensing. This can be attributed to the increase in conductive channels. However, at this stage, the nanomaterials within the lower conductive layer start to disconnect, causing a rise in tunnel resistance between neighboring MLG sheets. The upper conductive layer is also disconnected as a result of stretch strains, resulting in partial composite material slips, which significantly decreases the conductive channels. This results in a significant rise in the relative resistance change at large strains after 300%. Furthermore, the sensing range of the sensor was narrowest without MLG filling inside the flexible substrate, suggesting that the MLG-Ecoflex flexible substrate improved the sensing range. Nevertheless, the sensing range of the sensor with a concentration of 5 wt% did not demonstrate a noteworthy increase in comparison to the 3 wt%. This phenomenon resulted from the excessive filling of MLG, which heightened the stiffness of the flexible substrate, reducing its mechanical properties. 

The study in Figure 3c examined how variations in the mass ratios of the upper conducting layer MLG to CNTs composites affected the sensing curves. The results indicated that the slope of the curve of relative resistance versus strain increased first when the MLG to CNT mass ratio was 9:1, followed by 4:1, and 2:1, in that order, within the sensing range. The study indicates that the sensing effective strain range rises as the MLG to CNT mass ratio decreases and that heightened CNT inclusion enhances the sensor’s stretch properties. As the proportion of MLG increases, the amount of relative resistance changes and sensitivity increases, but the sensing range decreases. When close to the upper limit of sensing, the sensor’s resistance will increase steeply, so that the sensor is nearing open circuit. Figure 3d displays the relative resistive-strain variation curves of the strain sensor under different conductive material qualities. The sensor’s sensitivity increases with decreased conductive layer content, but the sensing range decreases. This is because of the lower conductive layer mass, which may cause the conductive channels to break at smaller stretch strains, resulting in higher sensitivity.

To summarize, the experiment at a substrate MLG concentration of 5 wt%, MLG:CNT ratio of 4:1 and the content of the upper conductive layer at 0.75 mg/cm^2^ yielded the best performing sensors with high stretchability (up to 580%) and excellent sensitivity (GF_max_ = 1517.94) (Figure 3e). The considerable increase in GF could be associated with the disconnection mechanism of MLG-CNT composites and the alteration in tunneling effect of Ecoflex polymers mixed with MLG. Compared to the reported research work (Figure 3f), it is demonstrated that the sensors in this work possess excellent strain sensing abilities with high sensitivity and wide operating range [11,13,20,24,25,29,30,31,32,33,34].

**Figure 4:**
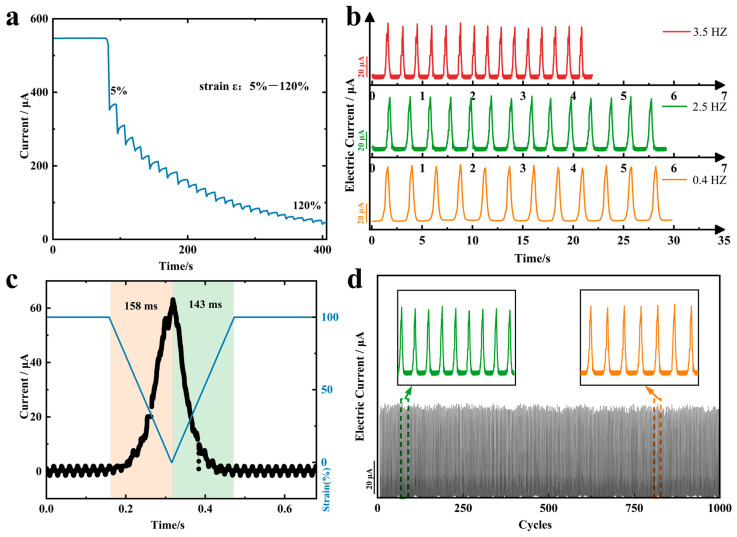
(**a**) Dynamic response of current signal for step strain in the range of 0–120% strain. (**b**) Variation of current signal of strain sensor at selected frequency at 100% strain. (**c**) Response time and relaxation time of strain sensor. (**d**) Cyclic durability curve of the strain sensor at 100% strain.

To evaluate the dependability of flexible strain sensors in this work, stretching experiments with different strain variables were carried out after the sensors were stabilized, as shown in Figure 4a, and the currents decreased accordingly as the amount of stretching increased. The frequency response of the sensor was tested at different stretching frequencies with the same strain variable (Figure 4b). The signal waveforms and heights at the chosen frequencies correspond with each other, indicating that the strain sensor has the ability to identify human motion at different frequencies. The current-time curves obtained during the tensile strain experiments showed that the strain sensors had response times of 158 ms (millisecond) and 143 ms for stress loading and unloading, respectively, at a tensile strain of 100% (Figure 4c). This remarkably short response times give the strain sensors the ability to operate under fast strains. We performed 1000 cyclic stretch tests under identical conditions, to determine the strain sensor’s repeatability and stability. The waveforms of each loading and unloading response signal were consistent and without significant degradation, signaling the sensors’ capability to maintain excellent performance during long-term reuse. 

### 3.3. Sensing Mechanism Analysis

To delve deeper into the potential mechanism behind the sensing properties of flexible strain sensors with bionic parallel vein-like structure based on a double-layer conductive network, the upper conductive layer’s surface morphology was characterized under different degrees of strain using scanning electron microscopy (SEM). 

**Figure 5:**
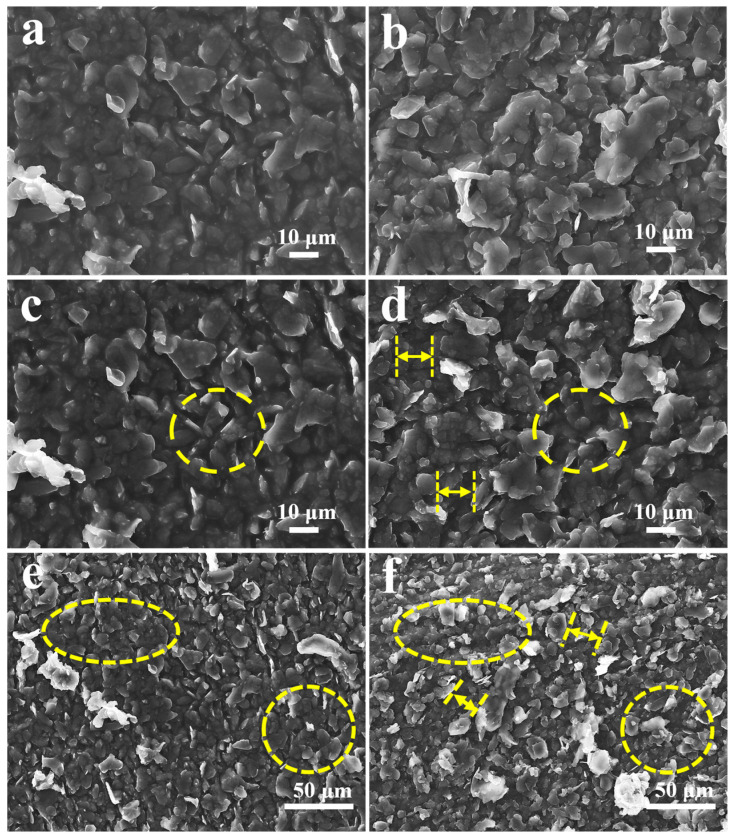
Characterization of the surface morphology of the conductive layer of the sensor before (**a**,**c**,**e**) and after (**b**,**d**,**f**) different degrees of stretching strain.

The surface morphology of the upper conductive layer under different stretching variables was characterized by scanning electron microscopy. It should be noted that the whitish part in the post-stretching SEM is a phenomenon due to the material sliding along the stretching direction, resulting in the disconnection of the conductive channel and inability to transmit the electron load. Inspection of the conductive layers before and after stretching at lower strains did not observe significant material slippage (Figure 5a,b), so the relative resistance values at this point changed little. However, as the degree of stretching strain grew, the upper layer experienced noteworthy material slippage, displaying a 10 μm (micrometer) slip distance on average (Figure 5c,d), which means a decrease in the overlapped region between the layers. At this point a small portion of the conductive channel was disconnected and the change in relative resistance value increased. Finally, at high levels of strain, the deformation of the conductive network increased, and a large number of flakes were observed in the upper out of contact conductive layer, with slip spacing reaching more than 30 µm (Figure 5e,f). At this stage, a significant number of disconnections in the conductive channels and a marked increase in the amount of change in relative resistance value resulted in a sudden rise in sensitivity during the large strain phase of the experiment. To summarize, in the unstretched state, the parallel vein-like microstructure of the substrate surface does not change significantly, and in the conductive layer consisting of composites of MLG-CNT, electrons can pass through the overlapping nanomaterials in the conductive network, and the resistance remains stable. When the sensor undergoes stretching stress, the substrate stretches and causes deformation of the surface microstructure. This leads to stretching of the connections between the interconnected pit structures and loss of overlap areas and electrical connections of some of the interconnected nanomaterials. As a consequence, the quantity of contact points and contact area within the conductive network established by the MLGs and CNTs on the upper conductive layer’s surface decreases, ultimately increasing the resistance. From the microstructural perspective, the disconnection of the overlapped nanomaterials under stretch occurs due to slippage caused by the weak interfacial bonding between the nanomaterials and the stretchable polymer, as well as the significant stiffness mismatch. After the release of strain, the elastomer undergoes a reverting motion, leading to the reconstruction of the conductive network in the conductive layer, which restores the microstructure of its substrate to its initial morphology. Consequently, the sensor resistance returns to its initial state. CNTs wrapped around the nanosheets have a bridging effect between the composites, and the CNTs in contact with each other effectively impede the complete disconnection of the conductive channel when the graphene sheet slides a large distance between the sheets. Therefore, the connection of the conductive channel can still be ensured under large strains, which greatly enhances the effective strain range. Furthermore, the conductive polymer composites acquired by mechanically blending MLG with the elastic polymer substance Ecoflex were used to prepare the lower conductive network. According to the tunneling effect theory [35,36,37,38], for this kind of resistive conductive polymer composite strain sensor, when the separation distance between the conductive nanomaterials in the flexible substrate is sufficiently small, the electrons can form a conductive channel through the tunneling effect. As the degree of strain rises, so does the tunneling distance, and, consequently, the sensing resistance also increases. The primary operating mechanism of MLG-Ecoflex polymer composite strain sensors under low strain is the alteration of resistance value caused by the tunneling effect. When subjected to high strain, the graphene in contact with itself disconnects, leading to a considerable reduction in conductive channel and a subsequent surge in resistance value of the sensors.

**Figure 6:**
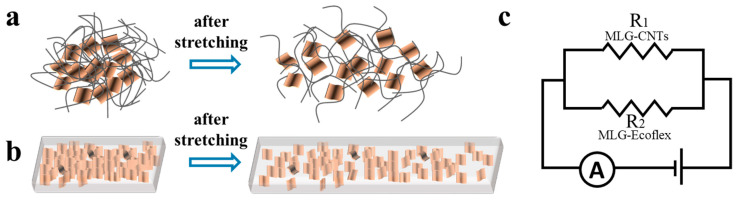
Schematic diagram of the (**a**) upper and (**b**) lower conductive layers before and after stretching and (**c**) a schematic diagram of parallel resistance.

Simply put, during the sensing process, the double-layer conductivity can be regarded as two resistors connected in parallel (see Figure 6c). Thus, the total resistance of the flexible strain sensors made of MLG-CNT/MLG-Ecoflex can be compared to two resistors connected in parallel, and the overall resistance can be calculated as $R=\frac{R_{1}R_{2}}{R_{1}+R_{2}}$. At low strain, it has much smaller resistance compared to the conductive polymer (*R*_2_) (i.e., *R*_1_ << *R*_2_), so that the conductivity is mainly influenced by the conductive layer of the composite material. As strain increases, the sliding disconnection between the overlapping materials and the resistance of the conductive layer of the composite material increases considerably. At this stage, the double-layer conductive coupling mechanism collaborates to impact the sensor’s conductivity. Compared to the flat structure, the deformation of the flexible substrate with a microstructured surface is primarily concentrated in the valley region during the stretching process. This concentration causes slip disconnection of the graphene sheet to be more prevalent in the flexible substrate. However, at this time, the CNTs surrounding the graphene sheet still provide the interconnection of the conductive pathway. Consequently, this double-layer conductive network coupling mechanism constitutes the high sensitivity and large operating range of the sensor.

### 3.4. Application of Human Motions Monitoring

**Figure 7:**
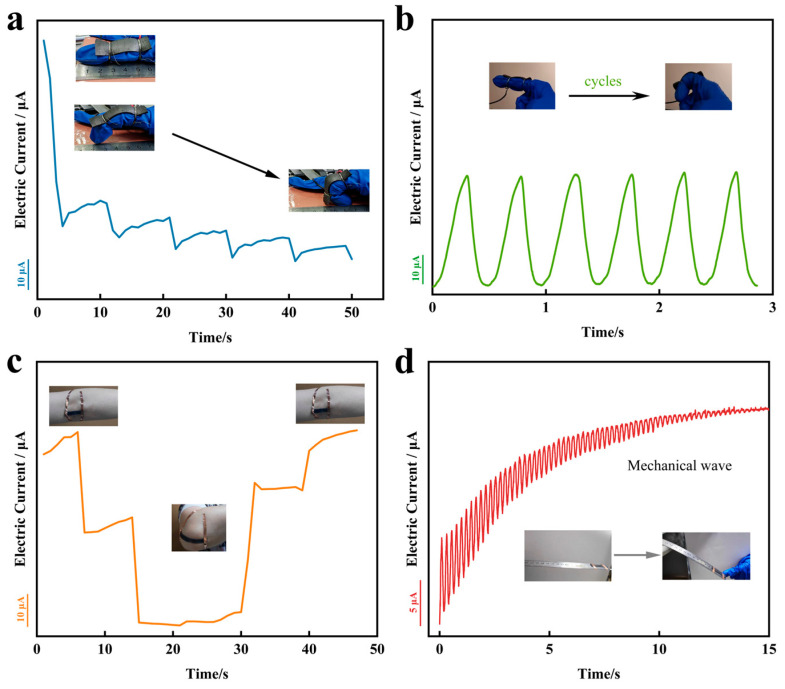
Applications for monitoring human movement ((**a**,**b**) the finger and (**c**) elbow joints). (**d**) Resonance motion monitoring of steel ruler.

When monitoring human movement or other practical applications, stretchable sensors need to be sensitive and repeatable enough under both large and small strains. The above experimental results show that the strain sensor has good linearity, excellent stretchable performance and high sensitivity in both small and large strain regions. In order to demonstrate the effectiveness of the sensor in human motion monitoring, the optimal sensor was attached to human joints to monitor the large deformations and small movements of the skin and muscles during finger and elbow joint movements. Different flexures of the finger and elbow joints resulted in different magnitudes of change in the current signal response (Figure 7a,c), and the magnitude of change in the current signal response to joint motion at the same flexure was consistent (Figure 7b). This indicates that the sensor is able to accurately respond to movements at human joints and has the potential to monitor the correctness of human locomotor movements. In addition, by attaching the sensor to a steel ruler and continuously monitoring the resonant motion of the ruler (Figure 7d) the sensor was able to monitor the changes in the mechanical waves in real time, which further demonstrates that the strain sensors in this work have excellent response time and sensitivity and are capable of a wide range of applications in the field of human sensing.

**Figure 8:**
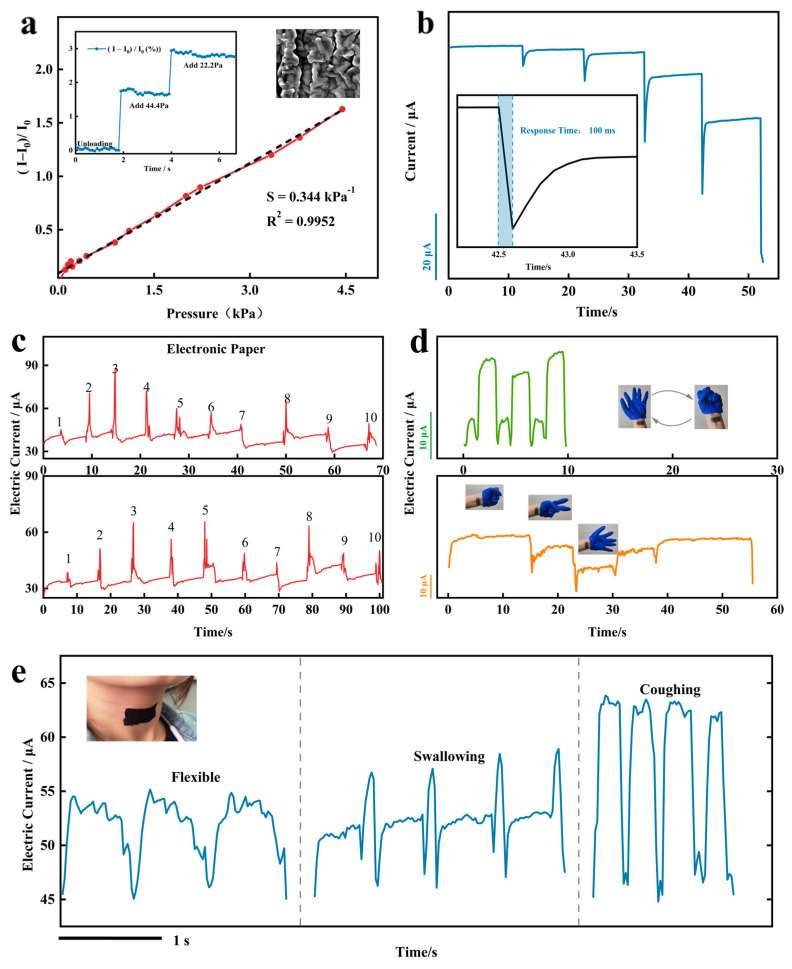
Pressure sensing curve of pressure sensitive sensor and its application. (**a**) Pressure-sensitive sensor response characteristics and (**b**) dynamic response curve. (**c**) Simulate e-paper applications. Application of monitoring (**d**) hand movements and (**e**) vocal vibrations.

Furthermore, following the coating of MLG-CNT composites on a flexible substrate to produce a conductive film, the two conductive films were oriented face-to-face and connected via silver adhesive electrodes and wires to generate a pressure-sensitive sensor. The pressure response curve is displayed in Figure 8a. The groove structure present on the parallel vein-like microstructure on the flexible substrate (Figure 2c) is able to deform the pressure-sensitive sensor when it is subjected to pressure, resulting in a rapid increase in the contact area of the conductive layer, which improves the sensitivity and sensing range of the sensor [39]. Referring to the previous experimental method for pressure sensing curve testing [40], the sensitivity can be defined as: $s=\frac{\Delta I∕I_{0}}{\Delta P}$, $\Delta I=I-I_{0}$; where *I* is the sensor’s current when the pressure is applied, *I*_0_ is the initial current when the sensor is not subjected to force, Δ*I* is the relative change of the current, and Δ*P* is the change in pressure. The inset in Figure 8a illustrates the alteration in relative current after sequentially increasing small intensity of pressures of 44.4 Pa and 22.2 Pa in sequence. It shows the ability of the sensor to respond at low strain range. The pressure response curve obtained through experimentation displays good linearity. After fitting, the sensitivity of the pressure-sensitive sensor is S = 0.344 kPa^−1^, and its response time is 100 ms (Figure 8b). The excellent sensing performance of this sensor is capable of accurately monitoring even smaller strains.

The size of the sensors in this work can be designed according to different applications; we prepared the sensors as 3 cm × 5 cm to simulate paper, wrote numbers 1 to 10 on them and monitored the electrical signals in real time. The signal response curves of both writings reveal similar peak shapes (Figure 8c), indicating stable performance of the pressure-sensitive sensor, which establishes its capability to record and differentiate written characters based on a characteristic waveform curve. Furthermore, we explored the application of monitoring the tiny movements of the human body. The pressure-sensitive sensor was attached to the wrist, and due to its excellent sensitivity, it was able to capture the subtle muscle movements induced by the fist-clenching and relaxation movements of the hand. The response curves were reproducible and stable during the fist-clenching and relaxation cycles, and the response to changes in the muscles and soft tissues of the inner wrist varies depending on the gesture made (Figure 8d). With further permutations and combinations, such as simultaneous monitoring of the wrist and finger joints, the sensor has the potential to achieve gesture recognition. We affixed a sensor outside the glottis and continuously monitored the vocal vibration and muscle movement of different words, including “flexible” vocalization and muscle movement during swallowing and coughing. The distinctions among the three response curves were noticeable, and the waveforms of individual movements were highly reproducible (Figure 8e). It is suggested that identification of each word or glottal movement can be accomplished through analysis of its distinct waveform, enabling greater convenience for those with aphasia. The pressure-sensitive sensor is highly accurate and repeatable in tracking even tiny movements, which can be utilized to develop more applications that bring convenience to humans.

## 4. Conclusions

A double-layer stretchable sensor using a flexible structured MLG-Ecoflex substrate and an MLG-CNT composite up-layer was developed in this paper. This sensor exhibits an excellent performance in terms of sensitivity (GF_max_ = 1517.94), extensibility (~up to 580%), linearity, stability (1000 stable cycles) and response speed (101ms). The sensing mechanism came from a slip disconnection mechanism in the composite up-layer and a tunneling effect in the low-layer together to enhance the sensitivity of the tensile strain sensor. In addition, a multilayered pressure sensor was also developed with a good sensitivity of S = 0.344 kPa^−1^. The capabilities of the two sensors used in human joint and muscle motion monitoring as well as audible sound monitoring were further demonstrated. It has the potential for use in such fields as medical monitoring, intelligent robotics and electronic skin, etc.

## Data Availability

All relevant data and information in this study are included in this article itself.
